# Role of Early Left Atrial Functional Decline in Predicting Cardiotoxicity in HER2 Positive Breast Cancer Patients Treated With *Trastuzumab*

**DOI:** 10.1007/s12012-024-09861-6

**Published:** 2024-05-02

**Authors:** Corinna Bergamini, Lorenzo Niro, Paolo Springhetti, Luisa Ferri, Laura Trento, Ilaria Minnucci, Caterina Maffeis, Elvin Tafciu, Andrea Rossi, Elena Fiorio, Giovanni Benfari, Flavio Ribichini

**Affiliations:** 1 Department of Medicine, Section of Cardiology, University of Verona, Piazzale Aristide Stefani 1, 37100 Verona, VR Italy; 2 Department of Medicine, Section of Oncology, University of Verona, 37100 Verona, VR Italy

**Keywords:** Trastuzumab, PALS, Cardiotoxicity, Breast cancer, CTRCD

## Abstract

Trastuzumab is widely used in HER2 breast cancer. However, it may cause left ventricular (LV) dysfunction. A decrease in LV global longitudinal strain (GLS) has been previously demonstrated to be a good predictor of subsequent cancer therapy related dysfunction (CTRCD). Left atrial morphological remodeling during Trastuzumab therapy has also been shown. The aim of this study is exploring the relationship between early changes in left atrial function and the development of Trastuzumab-induced cardiotoxicity. Consecutive patients with diagnosis of HER2+non-metastatic breast cancer treated with Trastuzumab were prospectively enrolled. A clinical, conventional, and advanced echocardiographic assessment was performed at baseline and every three months, until a one-year follow-up was reached. One-hundred-sixteen patients completed the 12 months follow-up, 10 (9%) cases of CTRCD were observed, all after the sixth month. GLS and LVEF significantly decreased in the CTRCD group at 6 months of follow-up, with an earlier (3 months) significant worsening in left atrial morpho-functional parameters. Systolic blood pressure, early peak atrial longitudinal strain (PALS), peak atrial contraction (PACS) and left atrial volume (LAVI) changes resulted independent predictors of CTRCD at multivariable logistic regression analysis. Moreover, early changes in PALS and PACS resulted good predictors of CTRCD development (AUC 0.85; *p* = 0.008, *p* < 0.001 and 0.77; *p *= 0.008, respectively). This prospective study emphasizes that the decline in PALS and PACS among trastuzumab-treated patients could possibly increase the accuracy in identifying future CTRCD in non-metastatic HER2 breast cancer cases, adding predictive value to conventional echocardiographic assessment.

## Introduction

Breast cancer is the most common type of malignancy diagnosed in women and it is the first cause of cancer death among females worldwide [1, 2]. Approximately 20% of all breast cancer overexpresses the human epidermal growth factor receptor 2 protein (HER2) [3]. Trastuzumab, a humanized monoclonal antibody that selectively binds to the extracellular domain of HER2 has changed the natural history of patients with HER2 positive breast cancer improving survival rate. Although well tolerated, Trastuzumab is associated with an increased risk of cardiotoxicity, in terms of left ventricular (LV) dysfunction, probably due to the block of the HER2 receptor expressed by adult cardiomyocytes [4].

In common practice, LV ejection fraction (LVEF) determined by echocardiography is widely used to identify cancer therapy–related cardiac dysfunction (CTRCD); however, LVEF reduction reflects advanced myocyte damage and mainly detects overt LV dysfunction, when it might be too late to reverse the clinical course of the ongoing changes. Furthermore, Cardiac Magnetic Resonance represents in this setting a promising field of research in early-cardiotoxicity detection, particularly through T1, T2, and extracellular volume mapping, providing structural information and myocardial tissue characterization. [5]

Evidence in support of the use of 2D speckle tracking echocardiography as a promising tool in the early detection of LV function changes are accumulating and a few possible parameters of early cardiac dysfunction have been proposed [6–8].

However, even if Global Longitudinal Strain (GLS) has been introduced in the current definition of CTRCD, its clinical implications in cardio-oncology are still not fully understood and remain a subject of debate especially in the setting of breast cancer [9, 10]. The recent ESC guidelines on cardioncology included a GLS drop of > 15% in their definition of cardiotoxicity. However, the 3-year analysis of the SUCCOUR trial has provided new insights into the evaluation of echocardiographic GLS in breast cancer patients undergoing chemotherapy, leading to a reconsideration of its role [9, 11].

Left atrium (LA) enlargement has been associated with a higher risk of cardiotoxicity in a fashion manner and left atrial remodeling has been related to CTRCD development independently from baseline left atrial volume [12, 13].

Moreover, in literature there is growing evidence that peak atrial longitudinal strain (PALS) decline is a sensitive parameter in different cardiac pathological entities that could possibly add prognostic values to conventional echocardiographic assessment [14–16]. However, little evidence is available concerning the role of precocious LA dysfunction as predictor of future LV impairment in patients undergoing chemotherapy [17, 18].

Aim of this monocentric prospective longitudinal study is to explore left atrial longitudinal strain imaging trend over time in relation to conventional echocardiographic parameters of LV dysfunction in patients affected by HER2 positive breast cancer; moreover, to investigate the eventual relationship between early changes in left atrial function and the development of Trastuzumab-induced cardiotoxicity.

## Methods

Consecutive patients with first diagnosis of non-metastatic breast cancer and overexpression of HER2, treated with Trastuzumab in an adjuvant or neoadjuvant setting between 2014 and 2021 and referring to the Oncology Department of our institute were prospectively enrolled in the study. Patients treated with an anthracycline-based regimen were also included. The exclusion criteria were presence of systolic dysfunction at baseline evaluation (LVEF < 50%), cardiomyopathy, previous myocardial infarction or congestive heart failure, more than mild valve disease, valve prosthesis, pacemaker devices, previous kidney or heart transplant, advanced chronic kidney disease (defined as the presence of an estimated glomerular filtration rate ≤ 30 mL/min by Cockcroft-Gault formula or hemodialysis), other tumoral disease. Furthermore, patients with poor image quality in whom a speckle tracking analysis would not have been sufficiently reliable were excluded.

The study protocol was approved by the Institutional Review Board of the University of Verona (1701 CESC), and it was conducted in accordance with the Declaration of Helsinki. All participants provided written informed consent.

Each patient underwent a full cardiological assessment comprehensive of a complete echocardiogram at baseline before starting anthracycline therapy, after 3 months at the beginning of trastuzumab administration and then every 3 months up to one year. CTRCD was defined as 10% or more absolute reduction in LVEF to a value below 53% from the baseline according to the scientific statement from the American Heart Association [19, 20] and to the clinical practice of the Oncology Department of our institute.

### Clinical Data

A comprehensive clinical evaluation was performed. Baseline oncological details, comorbidities, cardiovascular risk factors, medications, and oncological treatment regimens were collected.

#### Echocardiographic Evaluation

##### Conventional Echocardiography Assessment

All patients underwent complete transthoracic echocardiography using an EPIQ (CVX or 7C) or iE33 ultrasound system (Philips, Best, The Netherlands), equipped with an S5 or X5 transducer (1–5 MHz).

Simpson’s biplane method was used to assess indexed LV end diastolic (LVEDVI) and indexed LV end-systolic (LVESVI) volumes, LVEF and indexed left atrial volume (LAVI). Diastolic function was determined by measuring E/A ratio, E/E’ ratio, deceleration time (DT), left atrial volume and maximum tricuspid regurgitation (TR) velocity. Right chambers were evaluated by tricuspid annular plane systolic excursion (TAPSE), S’ velocity at tissue doppler imaging (S’-TDI), and systolic pulmonary arterial pressure (sPAP).

Valve function was assessed by color doppler, continuous-wave Doppler and pulsed wave Doppler using all the appropriate approaches. The LV outflow tract diameter (LVOT) was measured in mid-systole at the aortic valve annulus and the Stroke volume index (SVi) was then calculated with pulsed wave Doppler on left ventricular outflow tract in apical 5 chamber view and then divided by the body surface area [21].

##### Advanced Two-Dimensional (2D) Speckle Tracking Echocardiography (STE) Assessment

LA and LV function were also assessed by STE and values of LA strain and GLS and reported as absolute values.

For speckle-tracking echocardiographic analysis of LA and LV, 2D grayscale images were acquired at a frame rate of 40–80 frames per second and at least three consecutive cardiac cycles were recorded. The analysis of recordings was performed offline with a semi-automated acoustic tracking software (Tomtec-Arena ®, Imaging Systems GmbH).

Left ventricular strain was assessed from the apical 4, 3 and finally 2 chamber view and the mean GLS was additionally derived [22, 23].

**Fig. 1:**
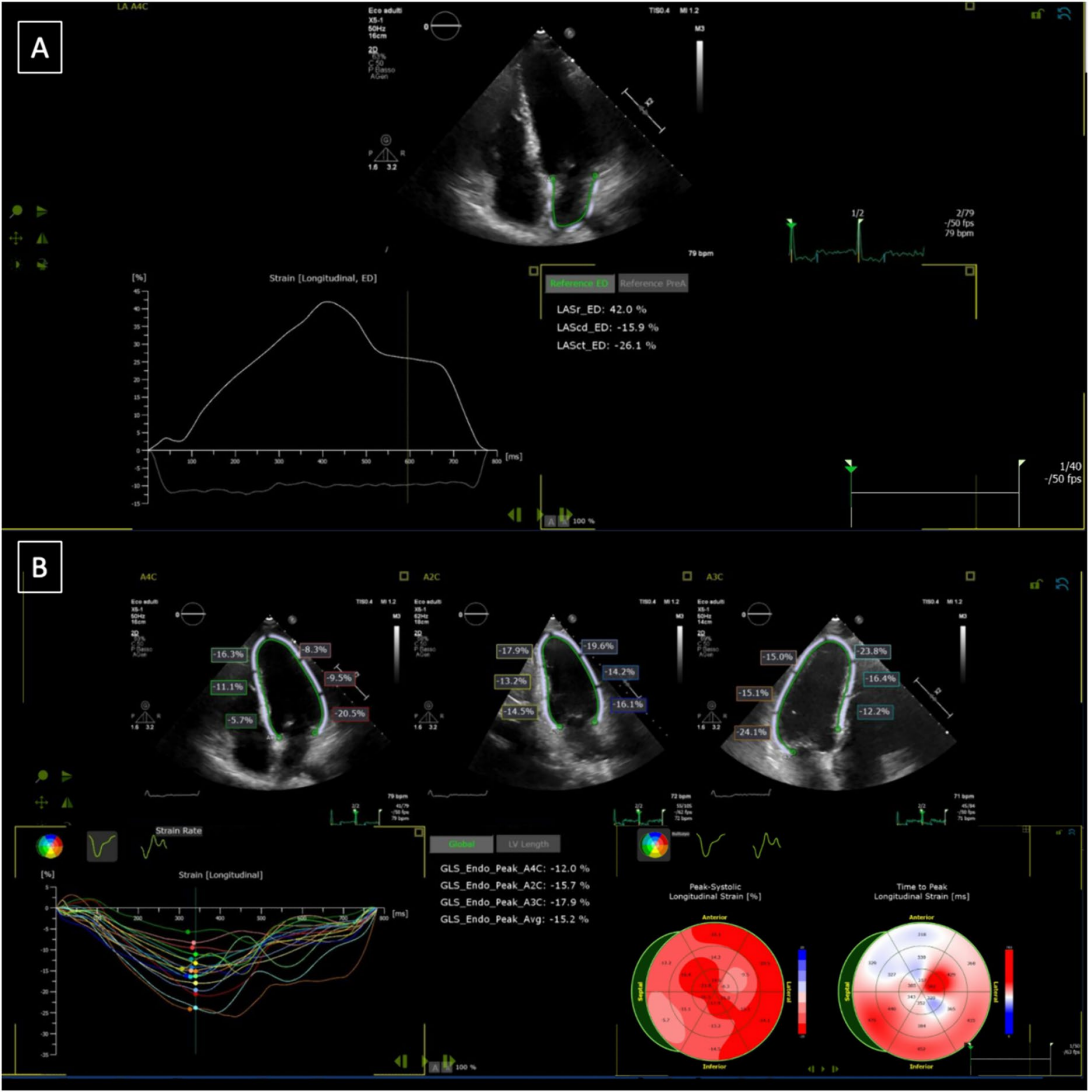
Example of LA (panel A) and LV (panel B) strain analysis

Left atrial strain was obtained from both the apical 2 and 4 chamber view and the mean value of PALS, Conduit (LACd) and Contraction (PACS) were then calculated tracking 3 points: medial mitral annulus, lateral mitral annulus and LA roof [24]. The region of interest was delineated by the Software and eventually optimized by manual adjustments when needed (Fig. 1).

To assess inter- and intra-observer variability 30 random patients were selected, and LA and LV strain analysis was performed in two different time points by the same investigator and a further analysis was performed by another investigator blinded to the previous results.

#### Statistical Analysis

Continuous variables are expressed as mean ± standard deviation, and categorical variables as absolute numbers and percentages when appropriate. Student’s t-test and analysis of variance (ANOVA) were used to compare normally distributed variables as appropriate, and the normality of the samples were assessed by the Shapiro–Wilk test.

Multiple linear regression analysis and logistic regression analysis were used to find eventual independent predictors of CTRCD. Only variables that were found to be significant in univariable analyses were included in multivariable analyses.

Trends of each echocardiographic variable during the treatment course were calculated by the following formula: [(GLS or LA strain values or LAVI at baseline – GLS or PALS or LA strain values at each follow-up) / GLS or LA strain values or LAVI at baseline].

Receiver operator characteristic (ROC) curve analysis was used to determine sensitivity and specificity of delta LA strain values 0–3 months and delta LAVI 0–3 months to predict upcoming CTRCD.

Intraclass correlation coefficient and coefficients of variation were used to assess inter and intra-observer variability. A two-tailed P-value < 0.05 was considered statistically significant.

Statistical analysis was performed by SPSS 25.0 software (SPSS, Inc., Chicago, IL, USA).

## Results

One-hundred-twenty female patients affected by non-metastatic HER2 positive breast cancer referring to the Oncology Department of our institute and receiving Trastuzumab treatment between 2014 and 2021 were enrolled in the present study. 116 patients completed the 12 months follow-up; 2 patients interrupted the protocol due to change of residence and 2 patients voluntarily withdrew from the study. 10 patients (9%) developed CTRCD during follow-up (CTRCD group), 106 (91%) did not (no-CTRCD group). Within those patients that showed a LV impairment during the treatment course, 7 developed CTRCD at 6 months and 3 at 9 months of follow-up. Only 2 (20%) patients that developed CTRCD were not previously exposed to anthracyclines.

### Baseline Clinical and Echocardiographic Characteristics

Clinical and echocardiographic characteristics of the population at baseline are listed in Tables 1 and 2.

**Table 1:** Baseline clinical parameters

	Overall	no-CTRCD group	CTRCD group	*P* value
Age	54 ± 13	54 ± 13	53 ± 14	0.62
BMI	24 ± 6	25 ± 6	28 ± 4	0.38
Systolic blood pressure (mmHg)	124 ± 17	123 ± 17	135 ± 14	0.03
Diastolic blood pressure (mmHg)	7 ± 12	73 ± 11	84 ± 15	0.01
Hypertension	23 (20.2%)	21 (20.2%)	2 (20%)	0.98
Dyslipidemia	28 (24.6%)	26 (25%)	2 (20%)	0.72
History of smoking	30 (26%)	28 (8%)	2 (20.0%)	0.65
Diabetes	6 (5.3%)	5 (4.8%)	1 (10%)	0.52
Familiarity for CAD	21 (18.4%)	18 (17.3%)	3 (30%)	0.35
ACE-I	8 (7.2%)	7 (7%)	1 (10%)	0.21
ARBs	5 (4.5%)	4 (4%)	1 (10%)	0.38
Beta-blockers	7 (6.3%)	6 (6%)	1 (10%)	0.24
Statins	10 (9%)	9 (8%)	1 (10%)	0.90
Taxane	116 (100%)	106 (100%)	10 (100%)	–
Anthracycline	93 (80%)	85 (80%)	8 (80%)	0.98
Radiotherapy	6 (5%)	5 (5%)	1 (10%)	0.51

*BMI* Body mass index, *CAD* Coronary artery disease, *ACE-I* ACE Inhibitors, *ARBs* Angiotensin receptor blockers

**Table 2:** Echocardiographic parameters at baseline

	Overall	no-CTRCD group	CTRCD group	*P* value
LVEDVI (ml/mq)	49 ± 7.0	49 ± 6.0	53 ± 8.0	0.04
LVESVI (ml/mq)	18 ± 3.0	18 ± 4.0	20 ± 4.0	0.13
LVEF (%)	63 ± 3.0	63 ± 3.0	62 ± 2.0	0.32
LAVI (ml/mq)	24 ± 6.0	25 ± 6.0	24 ± 5.0	0.59
E/A	1.1 ± 0.4	1.1 ± 0.4	1.0 ± 0.3	0.36
E’ medial (mitral) (cm/s)	9.4 ± 2.8	9.5 ± 2.7	8.6 ± 3.8	0.53
E’ lateral (mitral) (cm)	11.9 ± 3.7	11.9 ± 3.7	11.3 ± 3.1	0.59
dT (ms)	193 ± 54.0	193 ± 53.0	195 ± 70.0	0.92
e/e’	7 ± 2.0	7 ± 2.0	9 ± 4.0	0.41
Mitral regurgitation (mild)	49 (42%)	45 (43%)	4 (40%)	0.84
S’ TDI medial (mitral) (cm/s)	8.3 ± 1.6	8.4 ± 1.6	7.5 ± 1.4	0.11
TAPSE (mm)	24 ± 3.0	24 ± 3.0	24 ± 3.0	0.59
S’ (tricuspid) (cm/s)	13.3 ± 2.2	13.2 ± 2.2	13.5 ± 2.7	0.40
GLS (%)	20 ± 2.0	21 ± 2.0	20 ± 2.0	0.19
PALS (%)	46 ± 10.0	46 ± 11.0	46 ± 9.0	0.43
Conduit (%)	22 ± 7	25 ± 9	19 ± 7	0.037
PACS (%)	25 ± 7	22 ± 7	27 ± 8	0.05

*CTRCD* Cancer therapy related cardiac dysfunction, *LVEDVI* left ventricular end diastolic volume indexed, *LVESVI* left ventricular end diastolic volume indexed, *LVEF* left ventricular ejection fraction, *LA* left atrial, *dT* deceleration time, *TAPSE* tricuspidal annular plane systolic excursion, *GLS* global longitudinal strain, *PALS* peak atrial longitudinal strain, *PACS* peak atrial longitudinal strain

Mean age was 54 ± 13 years. Of note systolic and diastolic blood pressure (SBP and DBP) were higher in patients developing CTRCD compared to those who did not show systolic dysfunction during the follow-up (SBP 135 ± 14 vs 123 ± 17 mmHg; *p* = 0.03 and DBP 84 ± 15 vs 73 ± 11 mmHg; *p* = 0.01), while no other significant differences between the two groups of patients were shown. All patients received taxane chemotherapy. Among them, 93 patients (80%) also received anthracycline treatment. Additionally, a subset of 6 patients underwent radiotherapy (RT), and only 2 patients were diagnosed with paroxysmal atrial fibrillation.

Mean LVEDVI was 49 ± 7 ml/mq, mean LVEF was 63 ± 3%, mean LAVI was 24 ± 6 ml/mq. Mean GLS and mean PALS at baseline were 21 ± 2 and 46 ± 10%, respectively. Of note, PACS was significantly higher in the CTRCD group, while LACd was lower in the CTRCD group (27 ± 8 vs 22 ± 7, p = 0.05 and 19 ± 7 vs 25 ± 9, respectively).

Reliability of GLS and PALS measurements were assessed by intraclass correlation coefficient and by coefficients of variation on 30 random patients and resulted high for both the assessments. Intraclass correlation coefficient for intra-observer variability was 0.967 (95% CI 0.917–0.987, *p* < 0.001) for GLS and 0.964 (95% CI 0.923—0.983, *p* < 0.001) for PALS. Intraclass correlation coefficient for inter-observer variability was 0.945 (95% CI 0.884–0.974, *p* < 0.001) for GLS and 0.980 (95% CI 0.964–0.990, *p* < 0.001) for PALS. Coefficient of variation for GLS was 4.1% (95% CI 3.0–4.9) and 5.1% (95% CI 3.6–6.3) for PALS. Interestingly LVEDVI was significantly higher in patients in the CTRCD group compared to those in the no-CTRCD group (53 ± 8.0 ml vs 49 ± 6.0 ml; *p* = 0.04) even if still in the range of normality. No other significant differences were observed between the two groups.

### Echocardiographic Evaluation During Follow-up

#### Trends of LVEF

**Fig. 2:**
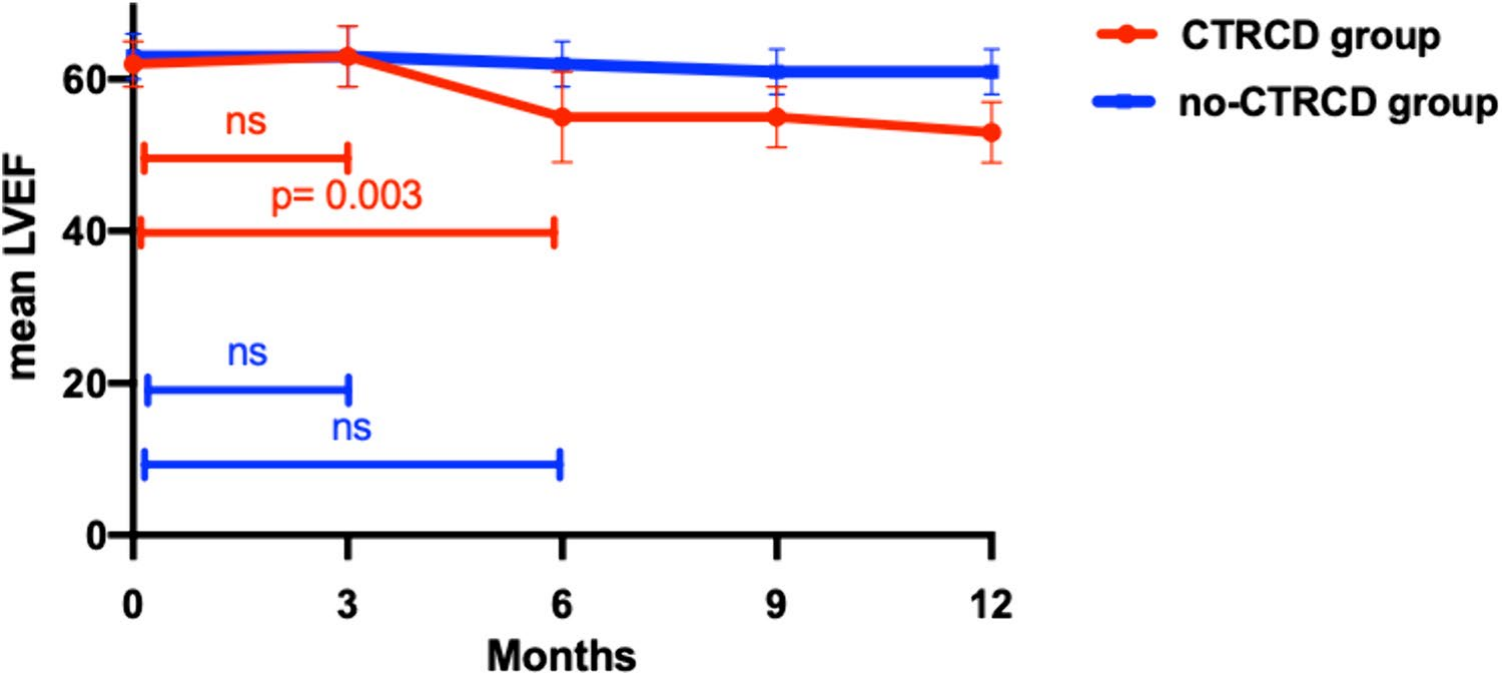
LVEF fluctuation in no-CTRCD and in CTRCD group

Figure 2 shows the fluctuation of LVEF during the treatment period in the no-CTRCD and in the CTRCD group.

During the first 3 months there was no significant reduction in LVEF compared to the baseline in both groups. Conversely, there was a significant decrease in LVEF after six months of treatment compared to the baseline only in the CTRCD group (62 ± 3 vs 55 ± 4%, *p* < 0.001).

The decline in LVEF continued significantly till the end of the follow-up in both groups; however, in the CTRCD group the reduction in LVEF was higher (delta LVEF 0–9 months 12 ± 7 vs 3 ± 7; *P* < 0.001 and delta LVEF 0–12 months 14 ± 6 vs 4 ± 6; p < 0.001 CTRCD group vs no CTRCD group, respectively).

#### Trends of LAVI

**Fig. 3:**
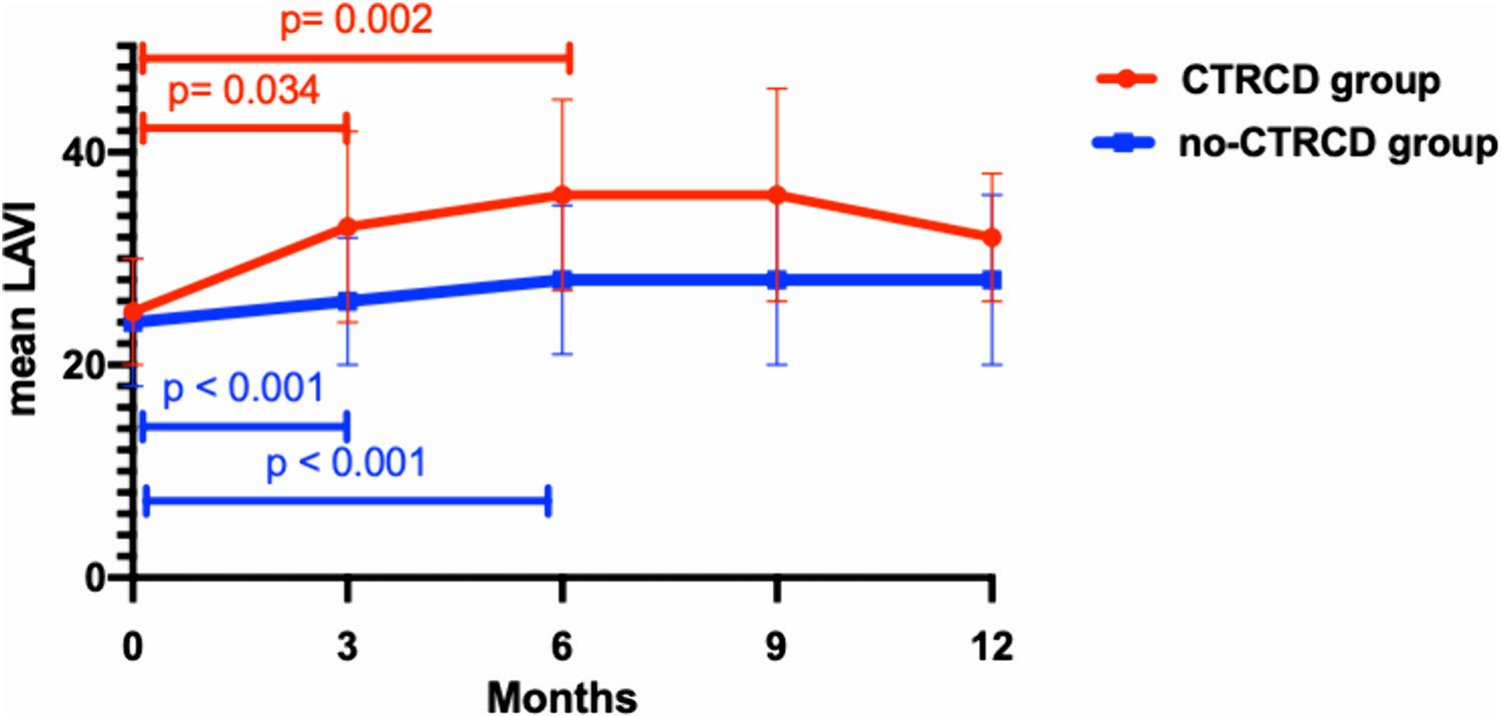
LAVI fluctuation in no-CTRCD and in CTRCD group

Figure 3 shows the course of LAVI during the follow-up. During the first 3 months of treatment there was a significant increment in LAVI in both groups. However, in the CTRCD group the atrial dilatation was significantly greater than in the no CTRCD group (8 ± 10 vs 2 ± 5%; *p* = 0.001). A similar trend was observed at six months of follow-up (11 ± 7 vs 4 ± 6%; *p* = 0.001)with a following plateau phase during the rest of the treatment period.

#### Trends of LV and LA Strain

**Fig. 4:**
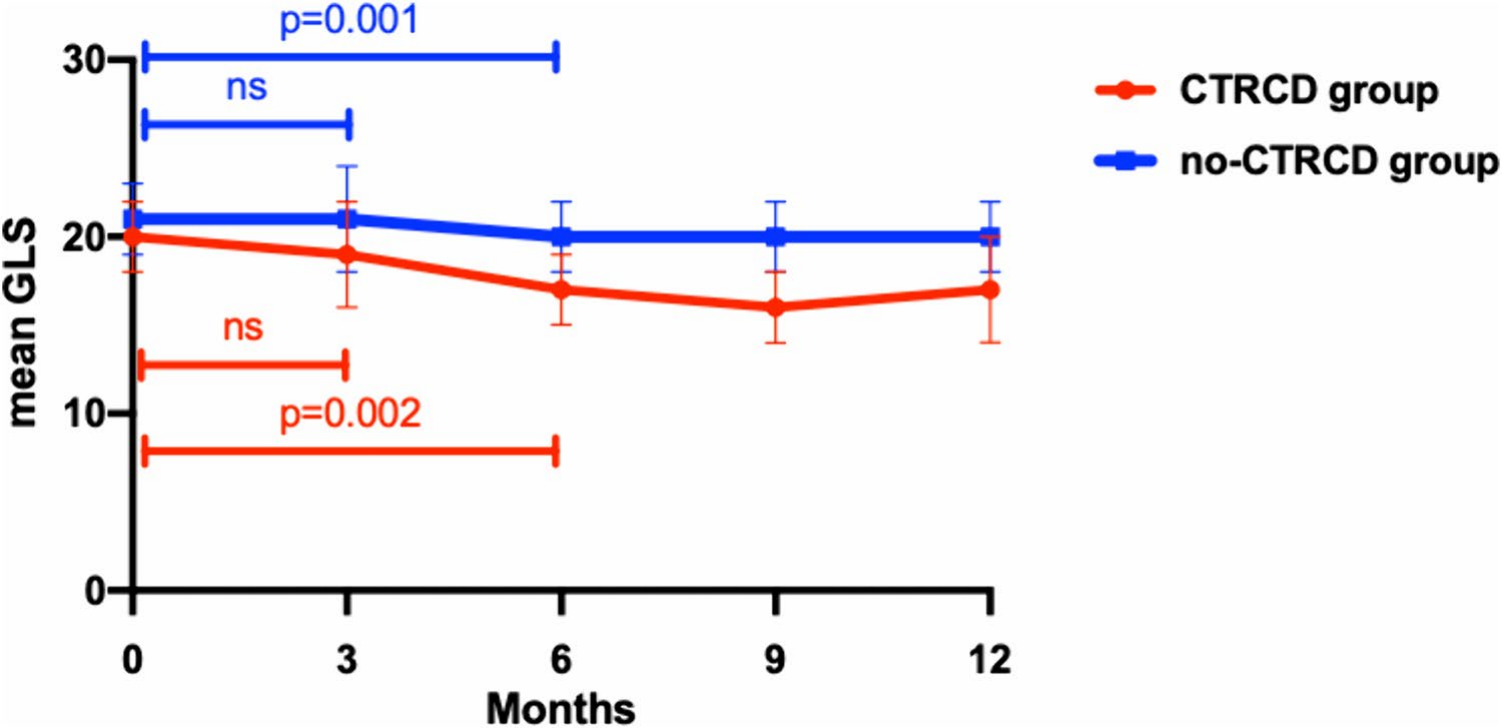
GLS fluctuation in no-CTRCD and in CTRCD group

Figure 4 shows the fluctuation of GLS during chemotherapy in the no-CTRCD and CTRCD group. During the first 3 months of treatment there was an initial reduction in GLS only in the CTRCD group, although not significant. On the contrary a significant decrease was observed in both groups at 6 months of follow-up showing a significantly greater reduction in the CTRCD group compared to the no-CTRCD group (delta GLS 0–6 months: 19 ± 9 vs 6 ± 13% *p* = 0.015, respectively). After 9 and 12 months of therapy, GLS remained significantly reduced compared to baseline in both groups, however at 9 months of follow-up such decrease was significantly higher in the CTRCD compared to the no-CTRCD group (delta GLS 0–9 months: 16 ± 7 vs 4 ± 13% *p* = 0.015, respectively).

**Fig. 5:**
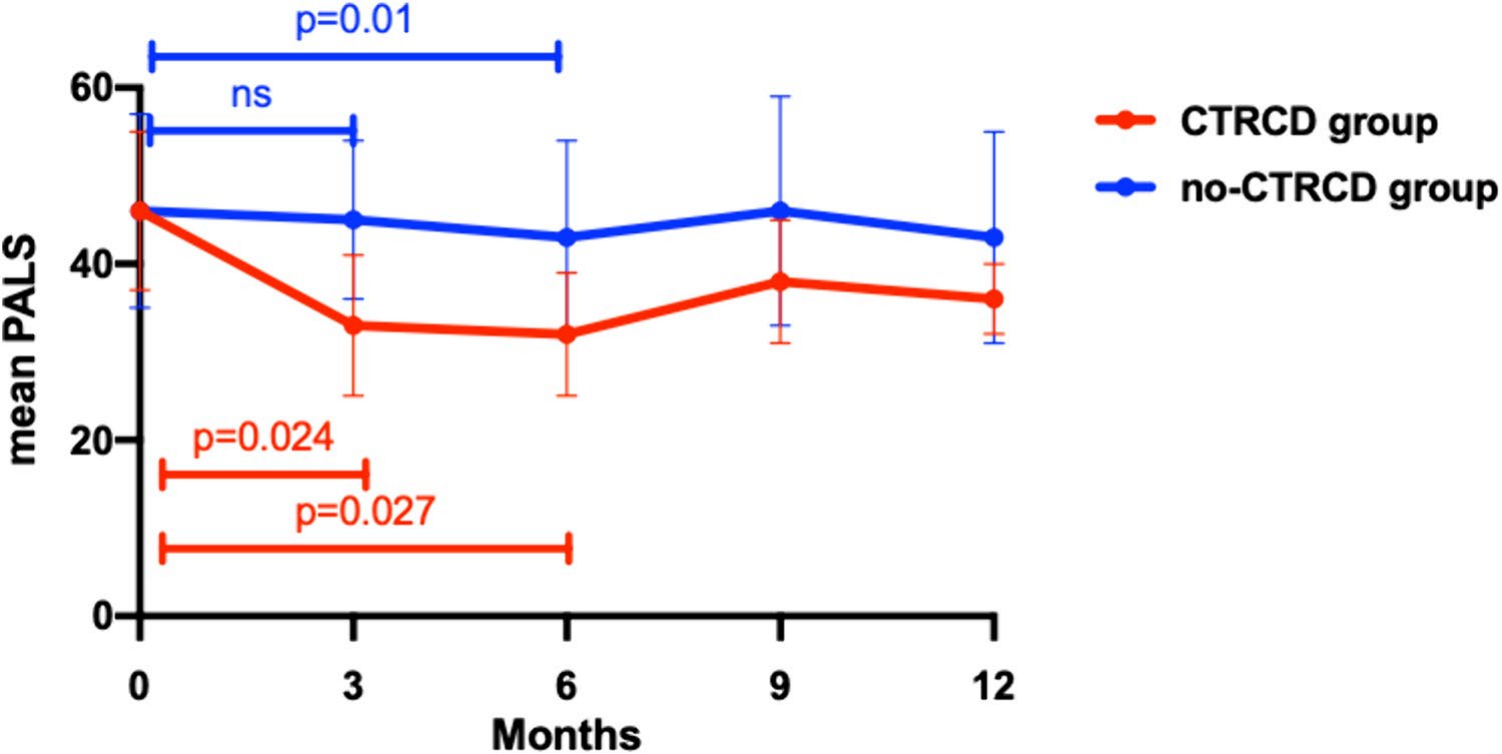
PALS fluctuation in no-CTRCD and in CTRCD group

Figure 5 shows the variation of PALS during chemotherapy in the two groups. During the first 3 months of treatment there was no significant reduction in PALS in the no-CTRCD group, on the contrary PALS significantly decreased in the CTRCD group (*p* = 0.024). Delta PALS 0–3 months was significantly different in no-CTRCD compared to CTRCD group (1 ± 2%, 21 ± 11%, respectively, *p* = 0.001).

After 6 months of treatment, both groups presented a significant reduction in LA strain, however in the CTRCD group the reduction was significantly greater than in the no-CTRCD group (delta PALS 0–6 months—30 ± 15% vs—4 ± 29%; *p* = 0.015).

**Fig. 6:**
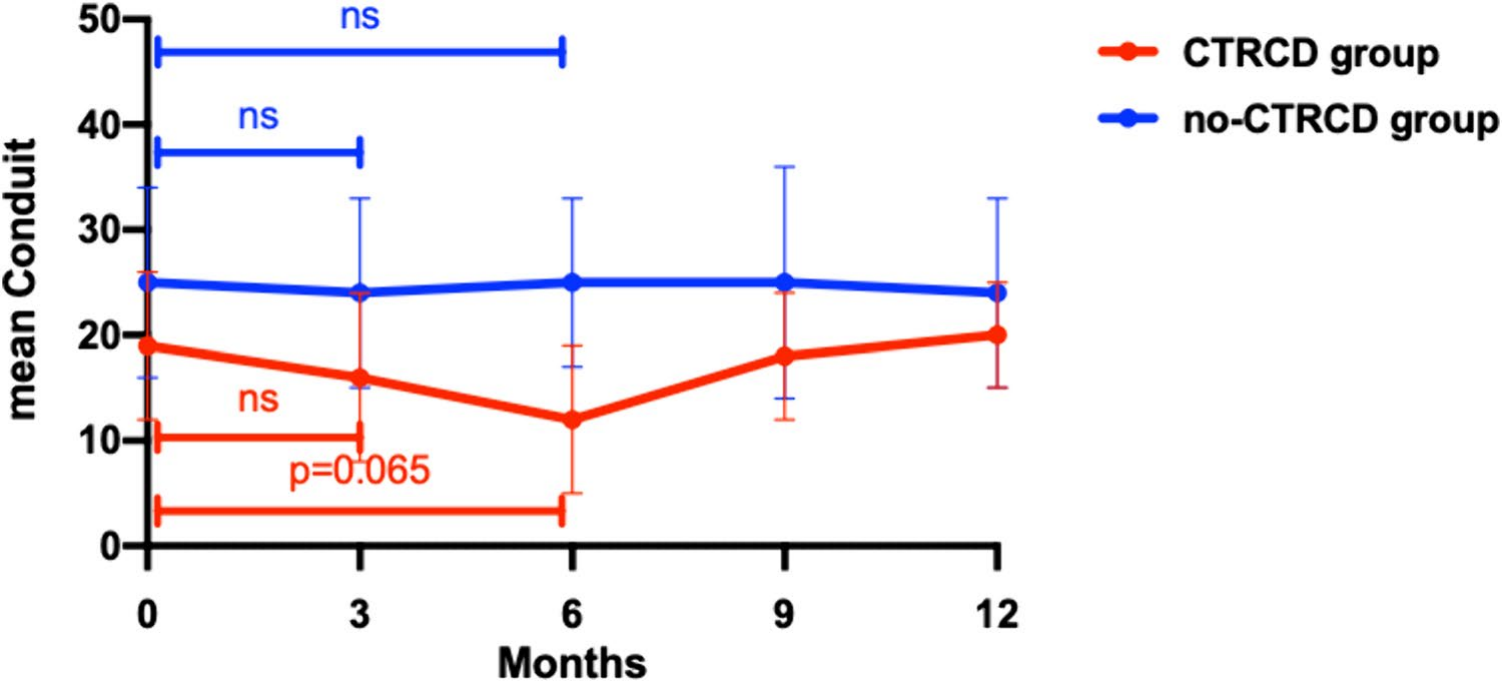
LACd fluctuation in no-CTRCD and in CTRCD group

**Fig. 7:**
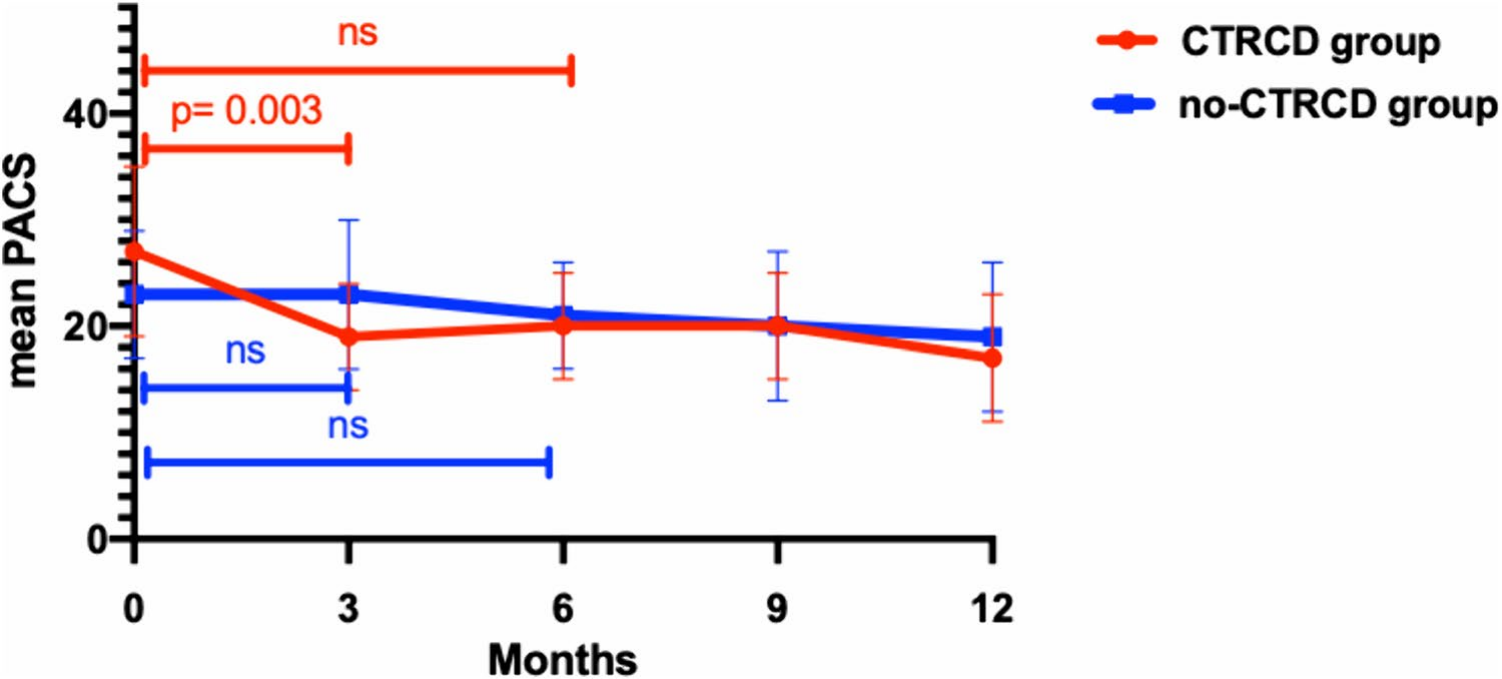
PACS fluctuation in no-CTRCD and in CTRCD group

Figures 6 and 7 show the trends of LACd and PACS, respectively. In the CTRCD group there was a significant reduction of PACs after three months of follow-up (delta PACS 0–3: 27 ± 16%, *p* = 0.003), conversely LACd didn't show a significant change. After 6 months of treatment, LACd presented a trend toward reduction even if not significant (delta LACd 0–6: 36 ± 42%, p = 0.065) in the CTRCD group, while PACS was not significantly different compared to baseline.

#### Predictors of Cardiotoxicity

Considering the trend of LAVI and LA speckle tracking analysis during the follow-up and their earlier changes compared to those of LV function, delta LAVI 0–3 months, delta PALS 0–3 months in addition to those clinical and echocardiographic variables that showed significant differences at baseline were tested in a univariable regression analysis.

SBP (*R*2 = 0.08, *p* = 0.04), LVEDVI (*R*2 = 0.12, *p* = 0.02), delta LAVI 0–3 months (*R*2 = 0.15, *p* = 0.01), delta PALS 0–3 months (*R*2 = 0.28, *p* = 0.003) and delta PACS 0–3 months (*R*2 0.15, *p* = 0.002) were found to be associated to the development of CTRCD (Table 3).

**Table 3:** Univariable analysis—CTRCD prediction

Variable	*R*2	*p* value
LEDVI (ml/mq)	0.12	0.02
Conduit (%)	0.06	0.05
PACS (%)	0.08	0.03
deltaLAVI (%)	0.15	0.01
deltaPALS (%)	0.28	0.003
deltaPACS (%)	0.15	0.002
SBP (mmHg)	0.08	0.04

*LVEDVI* left ventricular end diastolic volume indexed, *LAVI* left atrial volume indexed, *PALS* peak atrial longitudinal strain, *PACS* peak atrial contraction strain, *SBP* systolic blood pressure

Finally, because of the intrinsic strong association between the LA parameters, different separate multivariable logistic regression analysis including age and previous exposure to anthracycline were carried out to find the best predictors of cardiotoxicity (see Table 4).

**Table 4:** Logistic multivariable regression analysis (model 1, model 2, model 3 and model 4)—CTRCD prediction

Variable	H.R	C.I. 95%	*p* value
Model 1			
AGE	0.96	0.89–1.04	0.32
ANTHRACYCLINE	2.7	0.02–5.44	0.46
LVEDVi (ml/mq)	1.04	0.99–1.10	0.08
deltaLAVI 0–3 (%)	1.04	1.01–1.07	0.006
SBP (mmHg)	1.05	1.00–1.11	0.03
Model 2			
AGE	0.96	0.89–1.04	0.30
ANTHRACYCLINE	2.72	0.02–5.2	0.43
LVEDVi (ml/mq)	1.04	0.99–1.10	0.08
deltaPALS 0–3 (%)	1.18	1.05–1.34	0.008
SBP (mmHg)	1.07	1.01–1.16	0.037
Model 3			
AGE	0.96	0.89–1.04	0.38
ANTHRACYCLINE	1.32	0.6–9.8	0.83
LVEDVi (ml/mq)	1.05	0.98–1.11	0.08
deltaPACS 0–3 (%)	1.10	1.03–1.20	0.0001
SBP (mmHg)	1.10	1.02–1.19	0.002
Model 4			
AGE	0.98	0.91–1.06	0.76
ANTHRACYCLINE	1.69	0.06–6.2	0.65
LVEDVi (ml/mq)	1.03	0.98–1.09	0.19
PACS (%)	1.11	1.00–1.24	0.06
SBP (mmHg)	1.04	0.99–1.24	0.11

*HR* Hazard Ratio, *LVEDVI* left ventricular end diastolic volume indexed, *LAVI* left atrial volume indexed, *SBP* systolic blood pressure, *PALS* peak atrial longitudinal strain, *PACS* peak atrial contraction strain, *HR* Hazard Ratio, *CI* Confidence interval

DeltaLAVI 0–3 months, SBP at baseline and LVEDVi were included in Model 1. The first two parameters were found to be independent predictors of CTRCD (H.R 1.04 C.I 1.01–1.07 *p* = 0.006 and H.R 1.05 C.I 1.00–1.11 *p* = 0.03, respectively).

In Model 2 delta PALS 0–3 months and SBP at baseline were found to be independent predictors of cardiotoxicity (H.R 1.18 C.I 1.05–1.34 *p* = 0.008 and H.R 1.07 C.I 1.01–1.16 *p* = 0.037, respectively).

In Model 3 delta PACS 0–3 months and SBP at baseline were found to be independent predictors of CTRCD (H.R 1.10 C.I 1.03–1.20 *p* < 0.0001 and H.R 1.10 C.I 1.02–1.19 *p* = 0.002, respectively).

In model 4 baseline PACS presented a trend toward significance as an independent predictor of CTRCD (HR 1—1.24, *p* = 0.06).

**Fig. 8:**
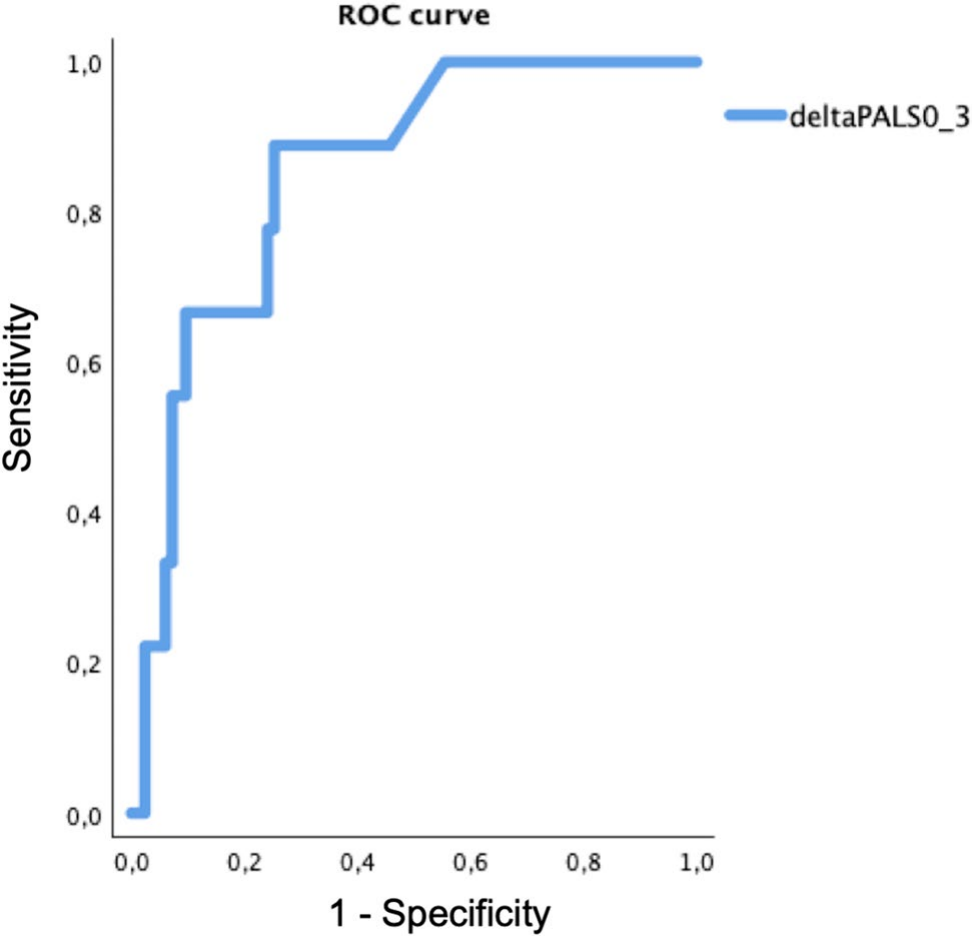
ROC curve for delta PALS 0–3 months in the CTRCD group

**Fig. 9:**
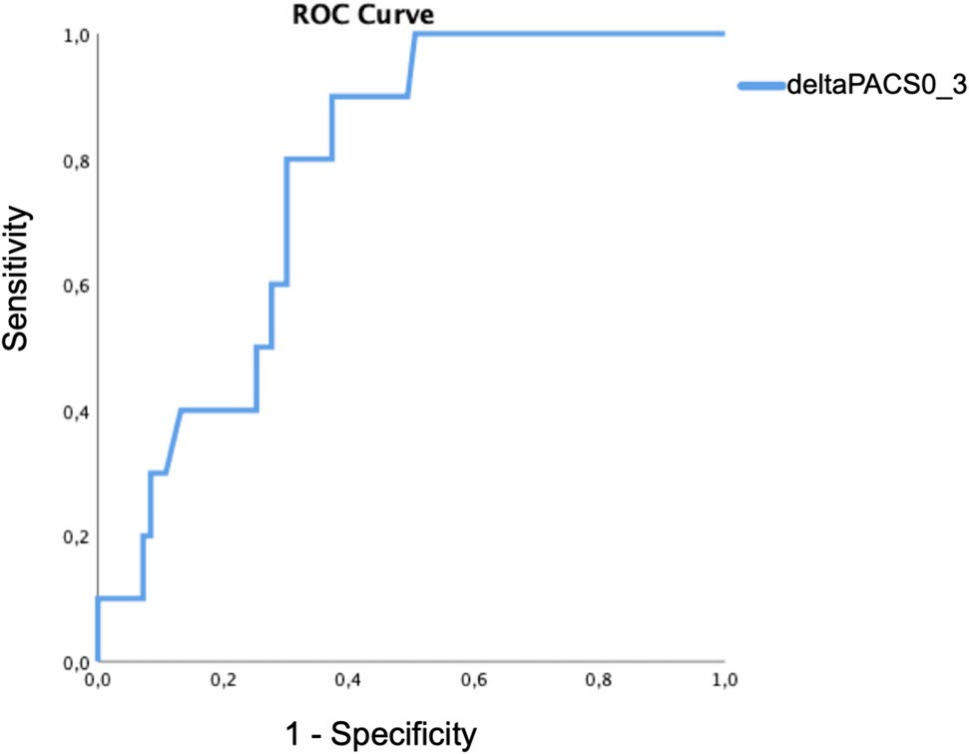
ROC curve for delta PACS 0–3 months in the CTRCD group

Moreover, ROC analysis showed that delta PALS 0–3 months (Fig. 8), and delta PACS 0–3 months (Fig. 9) presented a significative AUC in identifying CTRCD patients (AUC 0.85, *p* < 0.001 best cut off: 13% sensibility 89%, specificity 75% and AUC 0.77, *p* = 0.008, best cut off of − 14% sensibility 90%, specificity 62%, respectively), while delta LAVI 0–3 months did not, showing only a trend toward significance (AUC 0.710, p = 0.06, best cut off: 28% sensibility 60%, specificity 79%).

## Discussion

The major findings of the present study are as follows:

1. the development of cardiotoxicity in patients affected by HER2 positive breast cancer treated with Trastuzumab is not so rare and more importantly, patients developed CTRCD only after 6 or even 9 months of treatment.
2. Parameters of LV function significantly decreased after 6 months of follow-up in patients developing CTRCD compared to those not showing LV dysfunction; conversely, LA modification appeared after only 3 months of treatment. On one side the atrial size increased in both groups of patients although showing a greater enlargement in the CTRCD group while on the other side PALS and PACS showed a significant early drop only in patients who experienced CTRCD.
3. Systolic Blood Pressure at baseline, the early variation of LAVI, PALS and PACS during the first 3 months of treatment appeared to be independent predictors of cardiotoxicity.

Trastuzumab has changed the natural history of patients with HER2 positive breast cancer; however, it has documented cardiotoxic effects and the present study showed a prevalence of cardiotoxicity that appeared to be similar to that of the literature [25].

While LVEF fails to detect early damages of the myocardium, GLS is well known to be able to detect subclinical LV dysfunction before evident LV function decline in the CTRCD context [26]. Nevertheless, the present study shows that in HER2 positive breast cancer patients treated with Trastuzumab the LA appears to be the first chamber to be affected by the treatment.

Of note, a greater LAVI dilatation in CTRCD patients has been previously described in patients treated with Trastuzumab and former studies from our group highlighted that the LA enlargement was associated with a major risk of developing cardiotoxicity [11, 12, 27, 28].

Interestingly, LA strain showed a significant early drop only in patients who experienced CTRCD. Considering the limitations of the classic indexes of LA function, evaluation of LA deformation by advanced speckle tracking parameters has been a promising field of research in the last years. However, the prognostic role of PALS compared to GLS has been previously studied in different cardiac pathological entities with contradictory results [29].

The present study showed that PALS and PACS significantly dropped after 3 months of follow-up only in the CTRCD group, while a significant reduction in GLS was shown only after 6 months of treatment in both groups and with a significantly greater reduction in the CTRCD compared to the no-CTRCD group. Such findings are in line with those of Laufer-Perl et al. who showed a consistent reduction in LA strain values with no compatible change in GLS values in a population of patients undergoing anthracycline [30]. The impairment of LA strain was significantly higher also in patients who developed asymptomatic mild CTRCD in a very recent study by Di Lisi et al. [31].

In the current study at the univariable analysis the early changes in LA volume and function appeared to be associated with the future development of LV dysfunction in addition to the baseline Systolic Blood Pressure and LVEDVI. The latter was previously found to be an independent predictor of cardiotoxicity in a retrospective cohort study of patients treated with Trastuzumab [32]. However, when four separate models in multivariable analysis were performed, only delta PALS 0–3 months, delta PACS 0–3 months, delta LAVI 0–3 months and Systolic Blood Pressure at baseline were confirmed to be independent predictors of CTRCD.

The multivariable models considered Age and Anthracycline as additional variables in the analysis. Previous studies have shown that these factors, along with radiotherapy and cardiovascular risk factors, are significant predictors of CTRCD [33] [34]. Although there seems to be a higher prevalence of cardiovascular burden in CTRCD patients based on previous research, statistical significance was not achieved in the present study probably due to the relatively small number of CTRCD cases in an overall cohort with a low prevalence of comorbidities.

Hypertension has been widely recognized as a predictor of CTRCD development during Trastuzumab therapy [35]. In this study, although there was no statistical difference in the prevalence of hypertension history between the group of patients developing CTRCD and those without LV dysfunction during follow-up, baseline Systolic Blood Pressure emerged as an independent predictor of CTRCD. This suggests that LA strain variations may reflect associated hemodynamic changes, including elevated LV filling pressures. Consequently, the LA can be viewed as a valuable indicator of blood pressure status.

Moreover, the higher PACS and lower LACd at baseline in the CTRCD group suggest that these patients may have subclinical alterations of the LA before receiving HER2 inhibitors, with a relatively more hyperdynamic LA and increased atrial contribution to left ventricular filling during diastole.

Of note, only the AUC of delta PALS and PACS 0 – 3 months resulted significant, which may imply that LA functional changes may be a more sensitive marker for cardiac damage compared to the LA morphological variations. Moreover, in a recent study H. Park et al. demonstrated that PALS early decline showed better sensitivity and specificity in predicting future CTRCD than GLS decline in patients treated with Trastuzumab after chemotherapy completion [29].

To the best of our knowledge this is the first study showing that early LA strain decline is a predictor of CTRCD, preceding fluctuation of GLS.

Notably, some differences from the study by Park et al. [29] can be highlighted: (1) the prospective nature of the present study design; (2) comprehensive assessment of LA function. PALS, LAVI and other components of LA strain were examined; (3) the concordance trend of these parameters which strengthens the possible precocious effect of chemotherapy on LA function (4) the step of inter-variability testing which allowed consistency and robustness to the present findings.

According to these results, it is possible that atrial remodeling and dysfunction may arise from cardiotoxicity in a similar manner as ventricular dysfunction. The LA might be more sensitive than LV to chemotherapeutic agents because of different structural features, being LA more prone to hemodynamic alterations as well as to chemotherapeutic damage.

The reasons for LA chamber remodeling and dysfunction under the influence of Trastuzumab therapy can be seen either as a consequence of the chemotherapy-related ventricular damage and LV filling pressure elevation or as the direct effect of Trastuzumab itself on the LA myocardium fibers.

In this setting, an early subclinical LA function worsening seems to arise earlier than ventricular impairment and may anticipate more severe LV myocardial damage, reason why its early recognition and diagnosis may be the key for preventing later ventricular dysfunction, identifying patients at higher risk for future development of CTRCD. Furthermore, the 3-years analysis of SUCCOUR trial failed to demonstrate that a GLS-guided strategy compared to only LVEF could minimize CTRCD development after initiation of cardioprotective therapy, rising questions regarding the usefulness of LV strain and highlighting the need for other precocious markers [9].

## Limitations

This is a single-center observational study which included patients with only few cases of CTRCD. Furthermore, given the low prevalence of cardiovascular burden within our study population, the research was not adequately powered to investigate these specific clinical features in the context of CTRCD.

However, its strength is the prospective nature following a homogenous population and the fact that all echocardiographic exams have been performed by the same vendor and cardiologist, and the speckle tracking analysis was carried out by the same physicians and software.

Also, an echocardiographic follow-up after the conclusion of cancer therapy that could have been useful to investigate the irreversible or progressive nature of LA dimensions or functional changes, or even the possible reverse remodeling of LAVI in those long-term survivors patients, was not available. Furthermore, multi-imaging analysis is lacking; further research could investigate LA and LV structure with cardiac magnetic resonance in order to assess if PALS reduction and LA dilatation are eventually associated to ultrastructural changes.

Finally, cardiac biomarkers such as troponin and NT-proBNP were not always available so no specific further analysis was carried out to build a stronger predictive model of cardiotoxicity.

## Conclusion

This study highlights how Trastuzumab can cause both LA impairment and LV systolic dysfunction with different temporal trends. Of note, ventricular GLS worsening may be anticipated by the decline of PALS and PACS. Moreover, early changes of LA strain could possibly predict future development of CTRCD. Further studies are needed to investigate how the alterations in LA function and structure could be considered in an algorithm to detect patients who are prone to develop cardiotoxicity.

## Data Availability

All data relevant to the study are included in the article or uploaded as supplementary information.
